# Comparison of Pupil Dilation Responses to Unexpected Sounds in Monkeys and Humans

**DOI:** 10.3389/fpsyg.2021.754604

**Published:** 2021-12-23

**Authors:** Elena Selezneva, Michael Brosch, Sanchit Rathi, T. Vighneshvel, Nicole Wetzel

**Affiliations:** ^1^Research Group Neurocognitive Development, Leibniz Institute for Neurobiology, Magdeburg, Germany; ^2^Research Group Comparative Neuroscience, Leibniz Institute for Neurobiology, Magdeburg, Germany; ^3^Center for Behavioral Brain Sciences, Otto-von-Guericke University, Magdeburg, Germany; ^4^Department of Applied Human Sciences, Magdeburg-Stendal University of Applied Sciences, Magdeburg, Germany

**Keywords:** pupillometry, non-human primate, deviant, oddball, auditory

## Abstract

Pupil dilation in response to unexpected stimuli has been well documented in human as well as in non-human primates; however, this phenomenon has not been systematically compared between the species. This analogy is also crucial for the role of non-human primates as an animal model to investigate neural mechanisms underlying the processing of unexpected stimuli and their evoked pupil dilation response. To assess this qualitatively, we used an auditory oddball paradigm in which we presented subjects a sequence of the same sounds followed by occasional deviants while we measured their evoked pupil dilation response (PDR). We used deviants (a frequency deviant, a pink noise burst, a monkey vocalization and a whistle sound) which differed in the spectral composition and in their ability to induce arousal from the standard. Most deviants elicited a significant pupil dilation in both species with decreased peak latency and increased peak amplitude in monkeys compared to humans. A temporal Principal Component Analysis (PCA) revealed two components underlying the PDRs in both species. The early component is likely associated to the parasympathetic nervous system and the late component to the sympathetic nervous system, respectively. Taken together, the present study demonstrates a qualitative similarity between PDRs to unexpected auditory stimuli in macaque and human subjects suggesting that macaques can be a suitable model for investigating the neuronal bases of pupil dilation. However, the quantitative differences in PDRs between species need to be investigated in further comparative studies.

## Introduction

There has been a tremendous interest in pupillometry in the recent years as it offers insights into cognitive and emotional processes with a non-invasive approach. Pupil size alters not only in changing light conditions (so-called pupillary light reflex, PLR) but also in constant luminance condition. Also, pupil diameter (pupil dilation response, PDR) increases frequently during cognitive and emotional engagement (Kahneman and Beatty, 1966; Beatty, 1982), after an alerting or arousing stimulation (Hess and Polt, 1960; Bradley et al., 2008) and in response to unexpected stimuli (Friedman et al., 1973). Thus, PDR is commonly referred to as an index of arousal, cognitive load and attention-related processes (for a review see Sirois and Brisson, 2014; Mathôt, 2018; Zekveld et al., 2018). In monkeys, the PDR has been reported to be affected by arousal and cognitive processes such as an anticipated reward (Suzuki et al., 2016; Cash-Padgett et al., 2018), cognitive workload (Hampson et al., 2010), task conflict (Ebitz and Platt, 2015), decision confidence (Kawaguchi et al., 2018) and also with contrast-based visual stimulus saliency (Wang et al., 2014). In the auditory domain, the amplitude of PDR directly correlated to the spectral difference between stimuli in barn owls (Bala and Takahashi, 2000) and in guinea pigs (Montes-Lourido et al., 2021). Although using an animal model provides a way to understand the underlying neuronal mechanisms of pupil dilation, it is not yet clear how the PDR in animals (and especially in non-human primates) relates to that in humans. Thus, linking their PDRs has remained a central dogma not only for comparative neuroscience but also for systems neuroscience. Whereas the PLR along with its temporal dynamics are well inspected in rhesus monkeys (see Pong and Fuchs, 2000; Clarke et al., 2003), to our knowledge no such systematic study comparing PDRs between the monkeys and humans exists.

In animals, a positive correlation between pupil dilation and increased neuronal activity was reported in the locus coeruleus as well as in the superior and inferior colliculus (Joshi et al., 2016), in the frontal cortex (Hampson et al., 2010), in the anterior and posterior cingulate cortex (Ebitz and Platt, 2015; Joshi et al., 2016; Cash-Padgett et al., 2018) and in other cortical regions (for a review, see Peinkhofer et al., 2019). It is known that pupil dilation in humans occurs by inhibition of the parasympathetically innervated sphincter muscles and by activation of the sympathetically innervated dilator muscles (Mathôt, 2018), which has been discussed to be reflected by a biphasic PDR with two components (Steinhauer et al., 2004; Widmann et al., 2018). The first component peaks with a latency of 700 ms after stimulus onset reflecting the relaxation of the iris sphincter muscles whereas the second component peaks with a latency 1200 ms associated with the constriction of iris dilator muscles (Steinhauer and Hakerem, 1992). In one study, the application of pharmacological agents such as dapiprazole, which blocks the sympathetically mediated alpha-adrenergic receptor of the dilator, and tropicamide, which blocks muscarinic receptors in sphincter muscles, showed significant effects of lighting condition for the parasympathetic but not for the sympathetic pathway (Steinhauer et al., 2004). In an oddball study, Widmann et al. (2018) showed that only the first component, which is associated with the parasympathetic system, disappeared in darkness (since the sphincter muscle is maximally relaxed) and that only the second component, which is associated with the sympathetic system, was increased by highly emotionally arousing stimuli compared to moderately arousing neutral oddball sounds. In a lesion study, patients with ventromedial prefrontal damage showed smaller PDR during reward processing compared to healthy controls (Manohar and Husain, 2016). Simultaneous pupillometry and fMRI measurements during a visual oddball task revealed a correlation between pupil diameter and BOLD activity in the locus coeruleus (Murphy et al., 2014). Pupillary dilation was also correlated with fMRI activation in the ventral striatum and the globus pallidus during memory recording-retrieval tasks (Herweg et al., 2018).

To better understand the involvement of parasympathetic and sympathetic pathways of the autonomic nervous system in the PDR, we set out to directly compare the evoked PDRs by unexpected auditory stimuli in monkey and human subjects using the same experimental approach in both species. We adopted the auditory oddball paradigm previously used in a developmental study on pre-verbal children (Wetzel et al., 2016). We presented a simple harmonic tone as standard (500 Hz) and four deviant sounds, which differed in their spectral composition viz., a frequency deviant of 750 Hz, a pink noise, a whistle and a monkey vocalization, all of which were interleaved randomly within a sequence of repetitive standard sounds. We selected the “krahoo” call as a species-specific sound because longtail macaques can emit this call in “alarming” contexts, such that this call may potentially be an arousing auditory stimulus for conspecifics (Palombit, 1992). On the other hand, the whistle is a potentially less arousing “neutral” sound for both species.

## Materials and Methods

### Subjects

The data from five human subjects (four females, aged between 25 and 41 years with an average age of 30.6 years) and five macaque subjects (*Macaca fascicularis*; four males, age in the range of 10 and 18 years with a mean age of 13.8 years) were analyzed in the study. Three of the macaque subjects had previously participated in electrophysiological and behavioral experiments (Brosch et al., 2015; Knyazeva et al., 2020). The data from three additional macaque and three human subjects had to be excluded due to technical issues. All human participants confirmed having a normal or corrected-to-normal vision and normal hearing. They were naïve to the purpose of our experiment and gave written consent to a protocol approved by the local ethics committee. The animal experiments were approved by the authority for animal care and ethics of the federal state of Saxony-Anhalt (SB Tierschutz, Referat Verbraucherschutz, Veterinärangelegenheiten, Landesverwaltungsamt, Halle) and abided to the rules for animal experimentation of the European Communities Council Directive (86/609/EEC).

### Apparatus

The experiment was conducted within a double-walled soundproof room (1202-A; IAC, Niederkrüchten, Germany). Luminance in the room was kept identical for all participants at a level of ∼6 lx (Mavolux 5032B USB, Gossen Foto and Lichtmesstechnik GmbH, Germany). Monkeys were seated in a custom-made restraining primate chair in front of a laptop screen (ASUS, 17.3, with OS Linux 4.0.0). Additional head-restraining was attained by means of assembled units of 7 cm high plexiglass boards, which were placed around each monkey’s head such that their heads were oriented toward the screen. Human participants sat at the same position in an office chair with armrests. The distance between the screen and participants’ eyes was approximately 60 cm for both monkeys and humans. A silent animated cartoon film consisting of slowly to moderately moving animals, toys and colored geometrical figures was presented with the intention to draw the subjects’ attention and to keep their eyes within the eye-tracker’s range during data acquisition. The videos were presented at the center of screen with a width of 20 cm and a height of 10.8 cm, respectively. The median luminance of each video block was adjusted to 35 ± 1 cd/m^2^. Sounds were presented *via* two loudspeakers (Bose Companion 2 Series III Multimedia speaker system) located ∼50 cm beneath the laptop on each side to achieve the stereo effect. These sounds were uncorrelated to the occurrence of salient events in the movie. Pupil diameter was recorded with an infrared Eye-Link Portable Duo remote eye-tracker (SR Research Ltd., Canada; 500 Hz) placed on the bottom of the laptop screen. The experiment was designed using custom Matlab programs on Psychtoolbox 3.0.15 (Kleiner et al., 2007).

### Stimuli

**FIGURE 1:**
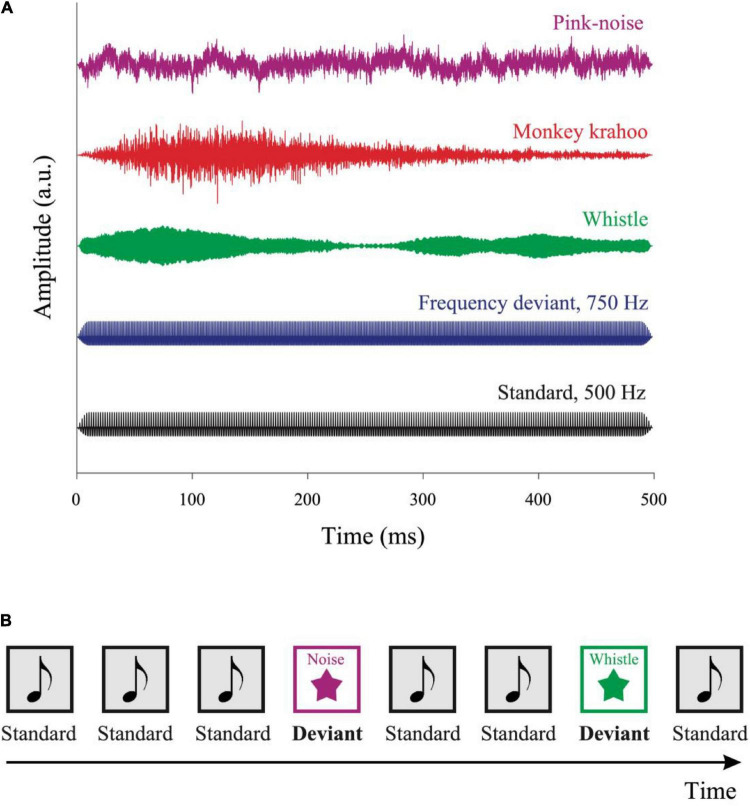
Auditory stimuli and the oddball paradigm. **(A)** Five auditory stimuli were used in the experiment: a standard tone, a frequency deviant, a whistle, a monkey “krahoo” and a pink noise-burst. Each sound was presented for 500 ms with a 10 ms rise/fall time at an intensity of ∼52 dB SPL. **(B)** A series of standard tones was presented in a pseudo-random order with occasional deviants. The sequence contained 80% of standard and 20% of deviant sounds, respectively, with at least two standards interleaved between consecutive deviants. The inter-tone interval was randomly varied between 2,700 and 3,300 ms.

Five different sound types were presented (see Figure 1A): a standard harmonic tone and four deviant sounds: a frequency deviant, a pink noise, a whistle and a monkey call. The standard harmonic tone, frequency deviant and pink noise were generated on Matlab. The whistle was collected from collaborative online database^1^. The monkey vocalization was a “krahoo” and was recorded in our monkey colony of the Leibniz Institute of Neurobiology. The fundamental frequency of the standard tone and the frequency deviant was 500 and 750 Hz, respectively. Both the tones consisted of the three lowest partials, the intensity of the second and third partials were lower than the first one by −3 and −6 dB, respectively. All sounds were presented with a duration of 500 ms including 10 ms rise/fall time and were root mean square (RMS) matched at an intensity of ∼52 dB SPL (measured with a PAA3 PHONIC Handheld audio analyzer, Phonic Corporation, Taipei, Taiwan).

### Procedure

Sounds were presented sequentially in an oddball paradigm consisting of 80% standard and 20% deviant sounds (see Figure 1B). A total of 200 sounds were presented in four blocks: 160 standard sounds, 10 frequency deviants, 10 pink noise bursts, 10 whistles, and 10 monkey calls. The sounds were presented in a pseudo-random order (different for each participant), with at least two standards preceded a deviant, and with no deviant followed by the same deviant type. The stimulus onset asynchrony varied randomly in 200 ms steps between 2,700 and 3,300 ms, with each SOA being presented at the same probability. The total duration of the four auditory blocks was ∼10 min. Subsequently, the tonic baseline pupil size (i.e., without auditory stimuli) was recorded for 3 min. Data recording started with a three-point eye tracking calibration procedure with a custom-made rotating visual target accompanied by a tone.

### Pupil Data Processing

In the first step, raw data from the eye tracker were converted into millimeters. Next, a series of post-processing was performed to exclude samples if they: (1) were marked as blinks by the eye tracker (values were rejected from the beginning of the corresponding saccade till its end); (2) were outside the physiological range (smaller than 1.5 mm or larger than 7 mm); (3) were disproportionately large change relative to their adjacent samples (e.g., samples with dilation speeds above the threshold calculated as the sum of median dilation speed and its median absolute deviation (MAD) which was then scaled by the factor of 32); (4) were outside the absolute trend-line deviations (identified by their abnormally large deviation from a smooth trend line). The latter two outliers i.e., the dilation speed and trend-line were detected using the Matlab toolbox described in Kret and Sjak-Shie, 2019. Additionally, the samples 50 ms before and after the gaps i.e., contiguous missing data sections larger than 75 ms were rejected from further analysis. Finally, the data samples generated only from both pupils were considered for data analyses. Gaps shorter than 700 ms were interpolated using the Matlab linear interpolation function *interp1*. The resulting mean data loss after interpolation was 5.6% in monkeys (range: 0.5–11.8%) and 0.2% in humans (range: 0.0–0.6%).

Individual average PDRs were computed from all segmented data epochs i.e., from 200 ms before till 1600 ms after sound onset for each of the five sounds separately and then the baseline was corrected by subtracting the mean amplitude during the initial 200 ms. We additionally calculated the relative changes in the pupil diameter by taking the ratio of the individual average PDR amplitudes through the post-stimulus epochs to the pre-stimulus period of 200 ms. The PDRs to the first two sounds in the block and the PDRs to the first two standard sounds after a deviant sound was presented were excluded due to the post-stimulation effects of the previous deviant sound. For the analysis on tonic pupil size, the pupil diameter recorded during the block with no auditory stimulation was averaged.

### Statistical Analyses

The PDR amplitudes of each sound type were averaged across repetitions (for the standard tone across ∼160 repetitions and for deviants across 10 repetitions each, respectively) along the time bins and then across the five macaque and the five human subjects. The mean PDRs were then compared to zero in a time-wise manner using student’s *t*-test at the α-level of 0.05 for at least ten consecutive points to determine the time windows in which there was a statistically significant response. The latencies of the maximal PDRs and PDR amplitudes around the peak (±50 ms) were calculated from both the individual subject PDRs as well as from the subject-averaged PDRs and then the latter were used for statistical analysis. To estimate the temporal response dynamics, we compared the latencies of PDRs obtained from four deviant types. The PDR amplitudes were analyzed using a two-way ANOVA with within-subject factor sound type (four deviant sounds) and with between-subject factor species (monkey vs. human). As the factor sound type had four levels, the Greenhouse-Geisser sphericity correction was applied. The eta square (η2) was used to estimate the effect size. Follow-up *t*-tests for independent samples were performed to compare the PDR amplitudes different sound types between species with an α-level of 0.05 and with a Bonferroni-corrected α-level of 0.0125.

Similar to the previous studies (Wetzel et al., 2016; Widmann et al., 2018), we used the ERP Principal Component Analysis Toolkit (temporal PCA Matlab toolbox described in Dien, 2010) to isolate the principal factors underlying PDRs. This toolkit is usually applied to the event related potential (ERP) data to achieve the identification of the constituent ERP components. We then applied the PCA to our PDR data set to investigate whether the PDRs of monkeys also consist of two components as it was previously described for humans (Steinhauer et al., 2004; Widmann et al., 2018). PCA was performed using a Geomin rotation with ε = 0.5 (Scharf and Nestler, 2019) and a covariance relationship matrix with no weighting. The number of components was determined using Horn’s parallel test. The PCA was conducted on individual averages for four deviant sounds (frequency deviant, pink noise, whistle, and monkey call) separately for each group, which resulted in a set of component loadings and a set of component scores. Component loadings were calculated to demonstrate the time-course of components meaning how much a component contributes during a specific time point. Thus, the component scores showed the standardized weights with which each principal component contributed to the observations. The original signal was then reconstructed as the sum of component loadings scaled by the product of component scores and their standard deviation (SD).

The PCA results were then analyzed using a three-way ANOVA with two within-subject factors viz., sound type (four deviant sounds) and component (early vs. late), and with the between-subject factor species (monkey vs. human). The Greenhouse-Geisser sphericity correction was applied to control for the sound type factor. To enable a comparison of PCA results for each sound type along the time course between monkeys and humans, components were computed by taking the product of component loadings, their standard deviation and the component scores (see Dien, 2010). We then calculated the mean amplitudes around the peak (±50 ms) of every temporal component. The three-way interaction was resolved using a follow-up two-way ANOVA with the factors sound type and species for the early and the late components separately. Follow-up *t*-tests were performed to compare the component scores of different sound types across species with a Bonferroni-corrected α-level of 0.0125.

## Results

### Tonic Pupil Size

The mean tonic pupil diameter, which was measured in the block without sounds, was 3.44 mm in monkeys and 3.66 mm in humans, respectively (see Table 1). There was no significant difference between the tonic pupil sizes across species (*t*(8) = 0.551, *p* = 0.597).

**TABLE 1:** Similar tonic pupil size in monkeys and humans.

Pupil diameter (mm)		
**Sr. No.**	**Monkeys**	**Humans**
1	3.57	4.64
2	4.06	3.89
3	3.42	3.69
4	2.60	3.48
5	3.56	2.62
Mean	3.44	3.66

*The pupil diameter is given for each monkey and human subject and their means.*

*It was measured during the baseline block i.e., with no auditory stimulation.*

### Pupil Dilation Response

**FIGURE 2:**
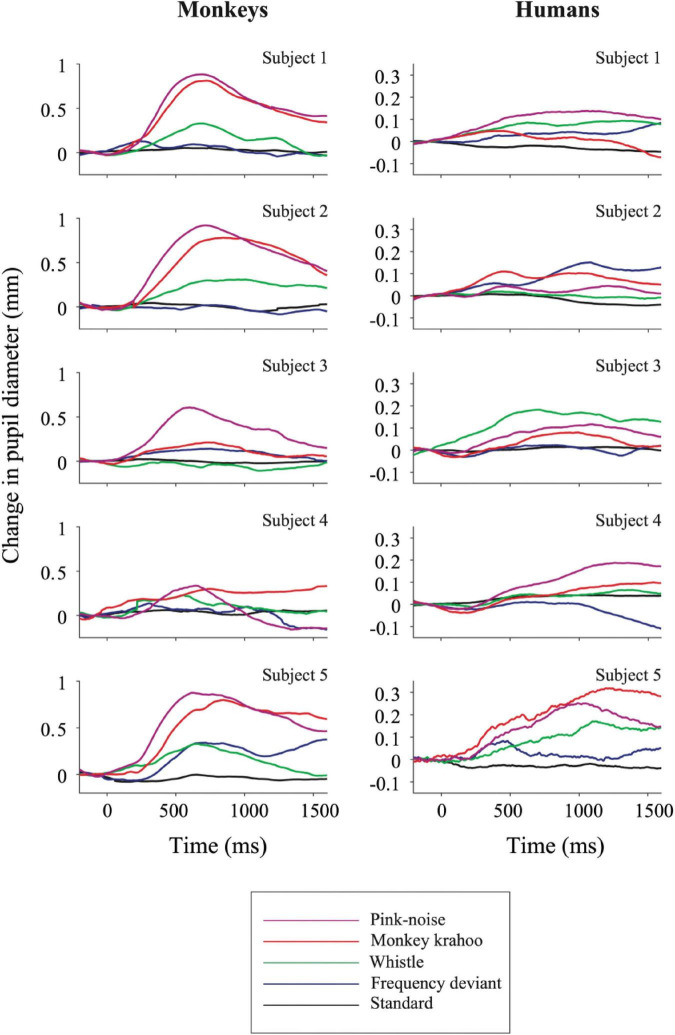
Auditory-evoked pupil dilation responses in individual monkey and human participants. Effects of standard and deviant sounds on PDRs are plotted against time for five monkeys (left column) and for five humans (right column). The activity was measured as a change in pupil diameter due to the tone onset with respect to its pre-onset period then averaged across repetitions. Positive values denote pupil dilation and negative values mean pupil constriction, respectively. Note the different *y*-axis ordinates between both species.

**FIGURE 3:**
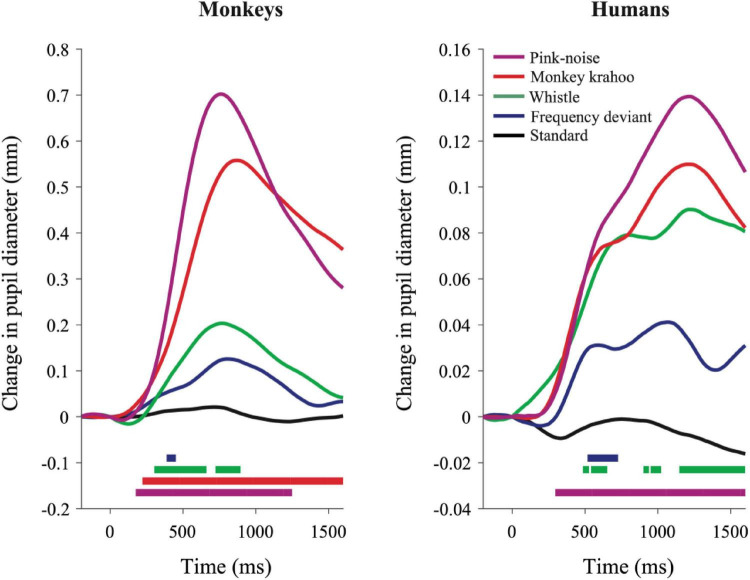
Qualitatively similar pupil dilation responses to sounds in monkeys and humans. Evoked PDRs for each tone are plotted as a function of time after averaging across tone repetitions and subjects (*n* = 5) in both species. Methodology and notations are same as in Figure 2. Horizontal colored bars at the bottom of each plot denote time bins in which the PDR was significantly different from zero (paired *t*-test, *P* < 0.05) during the time windows.

The individual PDRs of the five monkeys and the five human participants and their corresponding averages are shown in Figures 2, 3, respectively. Tables 2, 3 show values that were obtained from these plots. Most deviants elicited significant PDRs in both species. Since the occurrence of tones was not synchronized with the movie, average PDRs elicited by tone deviants in individual subjects were not confounded by pupil light reflexes. The PDRs in monkeys had a shorter latency and a higher amplitude compared to human participants. Pink noise, which had a maximal spectral bandwidth, elicited the strongest changes in pupil diameter. They started to be significant (e.g., p-level of the *t*-test was less than 0.05 for at least 10 consecutive points) at 176 ms in monkeys and 296 ms in humans and reached their maxima at 762 and 1218 ms, respectively. We used a *t*-test for independent samples and found that the average latency of the maximal PDR to deviant sounds was significantly shorter in monkeys than in humans (803 ms vs. 1180 ms, *t*(6) = 8.544, *p* = 1.4102e-04).

**TABLE 2:** Amplitudes and latencies of the maximal pupil dilation responses to four deviant sounds in monkey and human subjects.

Parameter	Species	Subject	Deviant sounds			
			Frequency deviant	Whistle	Monkey “krahoo”	Pink noise
Amplitude of maximal response (mm)	Monkey	1	0.13	0.33	0.81	0.88
		2	0.02	0.31	0.78	0.92
		3	0.14	0.01	0.21	0.61
		4	0.14	0.23	0.33	0.34
		5	0.40	0.33	0.80	0.87
Latency of maximal response (ms)		1	238	698	724	694
		2	794	958	848	714
		3	750	1726	746	606
		4	308	560	1602	642
		5	1682	654	846	624
Amplitude of maximal response (mm)	Human	1	0.09	0.09	0.05	0.14
		2	0.15	0.02	0.11	0.05
		3	0.03	0.18	0.08	0.12
		4	0.01	0.07	0.10	0.19
		5	0.08	0.17	0.32	0.25
Latency of maximal response (ms)		1	1794	1308	426	1056
		2	1084	440	444	1208
		3	1782	706	992	1098
		4	622	1382	1540	1260
		5	484	1120	1218	990

*Mean evoked-PDR of each subject was computed for every deviant sound across repetitions from the fragmented data epochs i.e., from 200 ms pre-stimulation till 1600 ms post-stimulation.*

**TABLE 3:** Mean pupil dilation response parameters for four deviant sounds in monkeys and humans.

Species	Parameter	Deviant sounds			
		Frequency deviant	Whistle	Monkey “krahoo”	Pink noise
Monkey	First latency (ms)	388	304	222	176
	Peak latency (ms)	808	770	870	762
	Peak amplitude (mm)	0.13	0.2	0.56	0.7
	Relative peak (%)	3.7	5.85	16.4	20.6
Human	First latency (ms)	518	486	–	296
	Peak latency (ms)	1070	1218	1214	1218
	Peak amplitude (mm)	0.04	0.09	0.11	0.14
	Relative peak (%)	1.0	2.6	3.6	4.1

*Evoked-PDR was computed from the grand averaged pupil response for every species and every deviant sound to estimate first latency, peak latency, and absolute and relative peak amplitudes of the PDRs. In general, monkey PDRs showed shorter first as well as peak latencies and larger peak amplitudes across all the deviants compared to human PDRs.*

To determine the effects of sound type on the PDR amplitudes in monkeys and humans, a two-way ANOVA with species and sound type as factors was performed. The ANOVA revealed significant main effects of the factors species (*F*(1,8) = 11.41, *p* = 0.010, η2 = 0.59) and sound type (*F*(1.81,14.46) = 13.35, *p* < 0.001, η2 = 0.22), thus reflecting that monkeys showed larger PDRs than humans and that different deviant sounds evoked different PDRs. The largest PDRs were obtained in the order of pink noise, monkey “krahoo,” whistle and frequency deviant. Note that a qualitatively similar order of response strengths was observed when responses were calculated relative to the 200-ms baseline level (see Table 2).

The interaction between the factors species × sound type (*F*(1.81,14.46) = 7.39, *p* = 0.007, η2 = 0.12) was also statistically significant. Follow-up *t*-tests (Bonferroni-corrected α-level of 0.0125) compared the PDR amplitudes in response to the different sound types between monkeys and humans. The amplitudes of maximal PDRs in monkeys were significantly larger than those in humans for the pink noise (*t*(8) = 3.611, *p* = 0.007), and barely missed the significance level for the monkey ‘krahoo’ (*t*(8) = 3.150, *p* = 0.014). No differences in the amplitudes of maximal PDRs between species were observed for the whistle (*t*(8) = 1.690, *p* = 0.130) and the frequency deviant (*t*(8) = 1.553, *p* = 0.159).

### Principal Components

**FIGURE 4:**
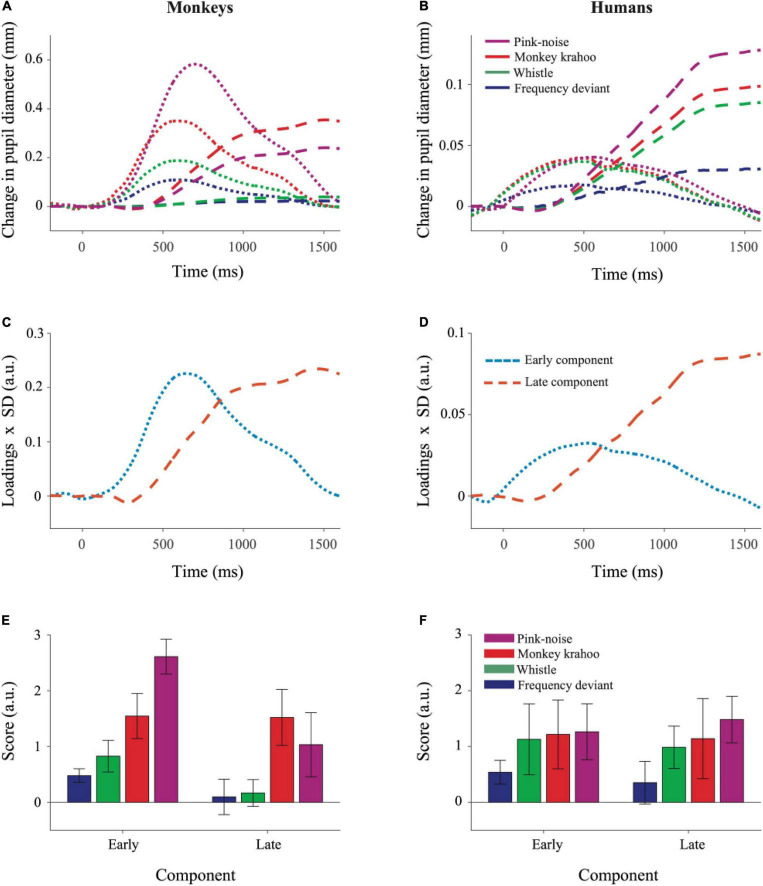
Principal Component Analysis illustrated similar pattern of component structure in monkey and human pupil dilation responses. The PCA was performed on mean PDRs across tone repetitions and subjects for each deviant group, which resulted in a set of principal component loadings and scores. **(A)** Represents reconstruction of monkey PDRs while **(B)** of human PDRs from PCA, wherein early and late component responses are depicted by dotted and dashed lines, respectively. Component scores were derived to determine the standardized weight with which each component contributed to the observations and were computed using the product of component loadings and their standard deviation (SD) across all deviants and their repetitions for monkey PDRs in **(C)** and for human PDRs in **(D)**. The aggregated early and late component scores over time for each deviant were averaged across tone repetitions and plotted for monkey PDRs in **(E)** and for human PDRs in **(F)**. Whiskers denote standard error of the mean (SEM) for the subject-averaged scores across tone repetitions.

The results of the Principal Component Analysis (PCA) are shown in Figure 4. PCA revealed similar temporal component structure in monkey and human PDRs. In both species, two components were obtained together explaining 97.8% (in monkeys) and 95.7% (in humans) of the variance. The early component had a peak latency of 604 ms in monkeys and 474 ms in humans and explained 41.0 and 15.4% of the variance in the two species. The late component had a peak latency of 1444 and 1552 ms (Figures 4C,D) and explained 56.8 and 80.3% of the variance in monkeys and humans, respectively.

To determine the effects of sound type on two components underlying the PDR in monkeys and humans, a three-way ANOVA with factors species, sound type, and component was performed. The ANOVA revealed significant main effects of the factor species (*F*(1,8) = 11.26, *p* = 0.010, η2 = 0.58) and of the factor sound type (*F*(2.13,17.05) = 12.78, *p* < 0.001, η2 = 0.19). The interaction between the factors species × sound type (*F*(2.13,17.05) = 7.63, *p* = 0.004, η2 = 0.11), species × component (*F*(1,8) = 13.50, *p* = 0.006, η2 = 0.06) and sound type × component (*F*(2.41,19.31) = 3.39, *p* = 0.047, η2 = 0.02) were statistically significant. The three-way interaction between the factors species × sound type × component was also statistically significant (*F*(2.41,19.31) = 5.52, *p* = 0.009, η2 = 0.03). A follow-up two-way ANOVA revealed significant interactions between the factors species × sound type for both the early (*F*(1.96,15.75) = 14.90, *p* < 0.001, η2 = 0.18) and the late (*F*(2.87,22.98) = 3.19, *p* = 0.044, η2 = 0.11) component. Then for each component, follow-up *t*-tests (Bonferroni-corrected α-level of 0.0125) compared the component scores in response to different sound types between monkeys and humans. The early component scores were significantly larger in monkeys than in humans for the pink noise (*t*(8) = 8.461, *p* = 2.9114e-05) and for the monkey “krahoo” (*t*(8) = 3.705, *p* = 0.006) but not for the whistle (*t*(8) = 2.491, *p* = 0.037) and the frequency deviant (*t*(8) = 3.135, *p* = 0.014; Figures 4E,F). No statistically significant differences between monkeys and humans were found for the late component scores (pink noise: *t*(8) = 0.900, *p* = 0.394; monkey ‘krahoo’: *t*(8) = 2.156, *p* = 0.063; whistle: *t*(8) = 0.802, *p* = 0.446; frequency deviant: *t*(8) = 0.107, *p* = 0.917).

To summarize, the pink noise which has a large contrast to the standard tone and to the potentially arousing monkey “krahoo” call evoked highest response in the early component in monkeys compared to humans while such an inter-species difference was absent for the whistle as well as for the frequency deviant. Such effects were not seen for the late component of the PDRs.

## Discussion

In the present auditory oddball study, we compared pupil dilation responses in human and non-human primates evoked by four unpredictable deviants viz., a frequency deviant, a whistle, a monkey call and a pink noise burst (Figure 1A), which were embedded in a sequence of standard sounds (Figure 1B). To estimate PDRs with comparable accuracy in both species, we limited the number of human subjects to be the same as the number of monkey subjects. Despite the small number, the characteristics of the human PDRs to unexpected sounds were similar to those we have obtained in previous studies with a sufficiently large number of participants (e.g., Wetzel et al., 2016; Bonmassar et al., 2020). In both species, the strongest PDR was elicited by a burst of pink-noise. In monkeys, we also observed a strong PDR to a species-specific vocalization, which suggests that PDRs in monkeys can also be driven by potentially arousing stimuli. These findings are in line with our previous developmental study with infants and adults wherein the largest PDRs were observed to the noise-burst and to an arousing deviant sound i.e., a baby cry, which were then followed by the phone’s ringtone and the frequency deviant (Wetzel et al., 2016). This pattern of similarity suggests that the monkey is a reasonable animal model to study the neuronal mechanisms underlying pupil dilation. In addition, the trend of PDRs within macaque subjects was found to be analogous to humans (Figures 2, 3), which once again emphasizes the value of non-human primates in invasive experiments with a limited number of subjects. Although we did not assess how much our monkey subjects attended the movie, it is unlikely that the observation of five-fold larger PDRs in monkeys than in humans can be fully explained by differences in attending the movie and the sounds. Studies in humans have shown that differences of PDRs between attended and unattended conditions are significantly smaller (see Zekveld et al., 2018 for review).

Overall, PDRs in monkeys were stronger compared to human PDRs. In contrast, no such differences were previously observed for the pupillary light reflex (PLR) as the strength of pupil constriction under different light conditions was reported to be quantitatively similar in both species (Gamlin et al., 1998, 2007). The latter indicates that differences in the PDR magnitude could not be simply due to differences in the pupil dynamic range between both species. A possible explanation could be provided by the neuroanatomic model of attention by Corbetta et al., 2008. The authors describe two separate cortico-cortical neural systems of attentional control: a dorsal frontoparietal network, which is biased for top-down signals and a ventral frontoparietal network, which is specialized for reorienting attention to new and behaviorally relevant stimuli and is linked to the locus coeruleus – norepinephrine system (LC-NE). The number of LC units is much larger in humans compared to macaques (45000–50000 vs. ∼10000) but is considerably lower than predicted according to allometric scaling relationship relative to neocortex and cerebellum volume (Sharma et al., 2010). This means that in monkeys, neocortex is supplied by more LC neurons compared to humans. The relative change in number of LC units in humans, however, correlates to the differences in brainstem’s medulla size between species. This anatomical dissimilarity may contribute to the observed diminished PDRs in humans compared to monkeys while the PLRs amplitudes remain compatible.

The LC-NE system controls the general arousal level (Carter et al., 2010) and is related to changes in pupil size (Aston-Jones and Cohen, 2005; Murphy et al., 2014; Joshi et al., 2016). Thus, stimulating the locus coeruleus increases extracellular noradrenaline concentration, which in turn inhibits only the spontaneous activity of sensory neurons but not the sensory-evoked responses. This net improvement in the signal-to-noise ratio can reduce detection thresholds and thus dramatically enhance perceptual acuity (for a review see Sara, 2009). Although the attentional engagement in the visual task cannot be exactly determined, potential differences in the allocation of attention to the sounds cannot explain the huge observed differences in PDR amplitudes. Also, the monkeys participating in our study were head-restrained so one could speculate that their overall level of arousal was higher compared to human participants. On the contrary, similar tonic pupil sizes were observed in both species reflecting the same level of general arousal and thus contradicting this speculation; however, to understand the exact relationship between absolute pupil size and arousal level is beyond the scope of our study and will be explored in future comparative studies. The difference in PDR magnitudes between both species could also result from different time courses of parasympathetic and sympathetic effects. In humans, the pupil size is regulated by two autonomic nervous systems: whereas the activation of parasympathetic nervous system causes pupil constriction in response to light stimuli, the activation of sympathetic nervous system causes pupil dilation due to a variety of arousing factors (for a review see Bouffard, 2019). In an attempt to delineate the influence of these two systems, we performed a PCA on PDRs which revealed two components with similar latencies in both species. The early component scores for the pink noise and the monkey call were larger in monkeys than in humans which was also reflected in shorter latencies of their PDR peaks. Such latency differences between monkeys and humans were previously reported for the PLR (Pong and Fuchs, 2000; Gamlin et al., 2007). In contrast to PLR, PDR is driven by the activation of muscles by the sympathetic system and by the inhibition of muscle activity by the parasympathetic system, respectively. The combined contribution of these two systems to pupil dilation has been discussed to produce a biphasic response in which the early component likely results from parasympathetic inhibition of the iris circular (sphincter) muscle and the late component results from sympathetic activation of the iris dilator muscle (Steinhauer and Hakerem, 1992; Widmann et al., 2018). In humans, an effect of emotional arousal was only observed for the late component, which is linked to the sympathetic system, but not for the early component (Widmann et al., 2018; Bonmassar et al., 2020). In line with this, Bradley et al., 2008 presented arousing (pleasant or unpleasant) pictures to human subjects and observed pupillary dilation in them which covaried with skin conductance but not with heart rate. They concluded that pupillary changes in response to emotionally arousing stimuli in human subjects are mediated by the sympathetic system alone.

Our results suggest a species-specific arousal effect (Palombit, 1992) due to the monkey “krahoo” call on both early as well as late components. The magnitude of the early component was higher in monkeys compared to humans. For the late component, this effect was also observed but barely missed statistical significance. The early component in monkeys also explained 41% of the variance compared to only 15% of the variance explained in humans. We suggest that the early peak in monkeys probably reflects the combined effect of both parasympathetic inhibition and sympathetic activation as the latter reaches its maximum ∼500 ms earlier compared to humans.

In sum, the present oddball study demonstrates striking similarities in the structure of temporal components underlying PDRs to unexpected sounds in monkeys and humans. Moreover, the noise-burst and the monkey vocalization caused pronounced pupil dilations in monkeys, similar to those observed in previous human studies (Liao et al., 2016; Wetzel et al., 2016; Widmann et al., 2018) demonstrating the specificity of the sound-related PDR. Thus, we consider the macaque to be a suitable model for invasive studies with an aim to understand the underlying neural mechanisms of PDR. However, the differences in amplitudes and latencies of PDRs between the two species as well as the different time courses of sympathetic and parasympathetic effects still need to be clarified in further comparative studies.

## Data Availability Statement

The raw data supporting the conclusions of this article will be made available by the authors, without undue reservation.

## Ethics Statement

The studies involving human participants were reviewed and approved by Ethikkommission der Otto-von-Guericke-Universität Magdeburg. The patients/participants provided their written informed consent to participate in this study. The animal study was reviewed and approved by the Landesverwaltungsamt Sachsen–Anhalt.

## Author Contributions

ES, MB, and NW conception and designed the experiment, and interpreted the results of experiments. ES performed the experiments and analyzed the data. ES and TV prepared the figures. ES, MB, SR, TV, and NW wrote the manuscript. All authors contributed to the article and approved the submitted version.

## Conflict of Interest

The authors declare that the research was conducted in the absence of any commercial or financial relationships that could be construed as a potential conflict of interest.

## Publisher’s Note

All claims expressed in this article are solely those of the authors and do not necessarily represent those of their affiliated organizations, or those of the publisher, the editors and the reviewers. Any product that may be evaluated in this article, or claim that may be made by its manufacturer, is not guaranteed or endorsed by the publisher.

## Footnotes

1
https://freesound.org/
